# Supplementary figures and images for: Uncovering a mitochondrial unfolded protein response in corals and its role in adapting to a changing world
Source: Proc Biol Sci. 2019 Jun 26;286(1905):20190470. doi: 10.1098/rspb.2019.0470 (PMC6599992; doi:10.1098/rspb.2019.0470)

A

| Query Sequence | Source sequence | Evalue |
|----------------|-----------------|--------|
| ATFS-1         | None            | NA     |
| Hs-ATF5        | <i>Of-ATF5</i>  | 6e-16  |

B

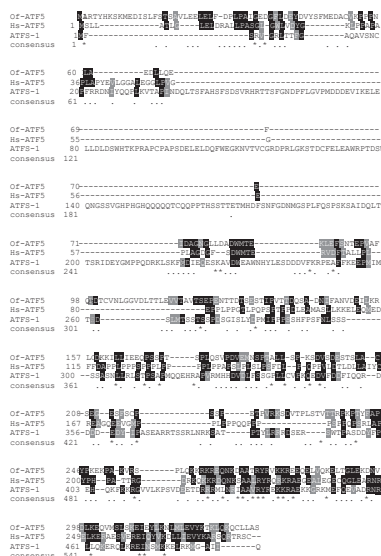

C

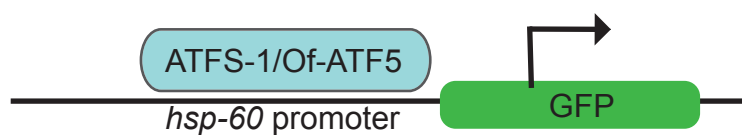

D

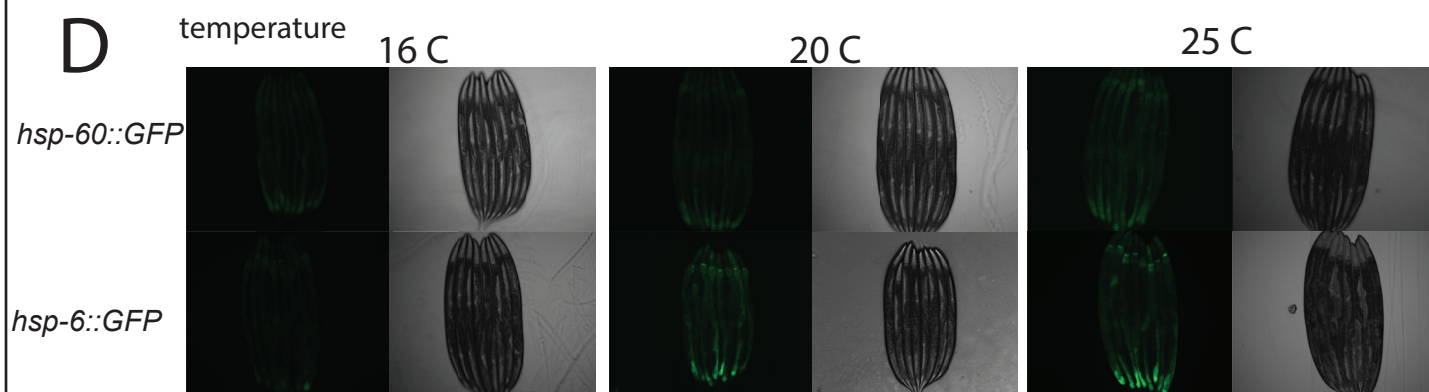

E

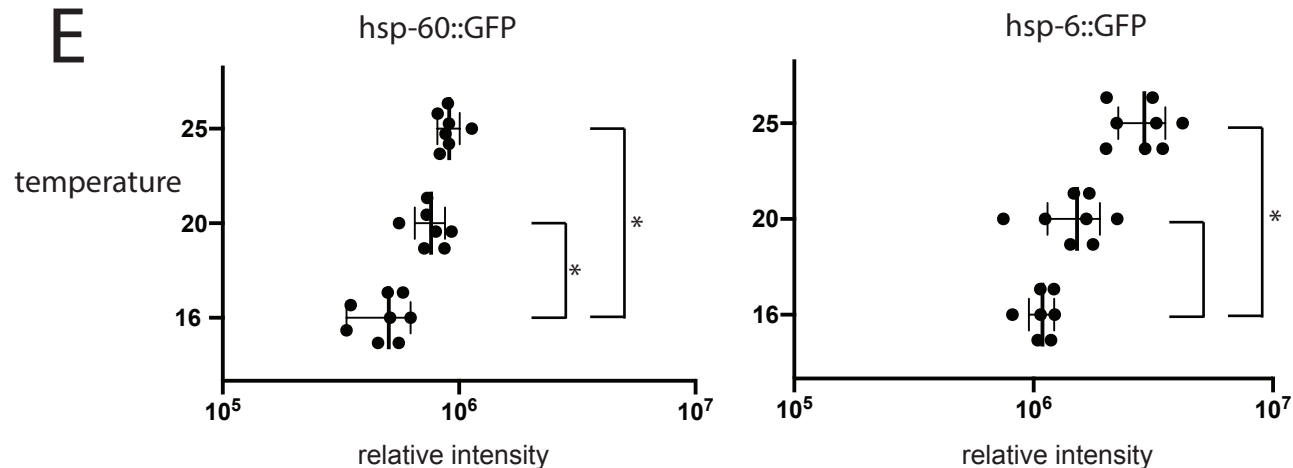

Supplement: Homology of UPRmt mediating proteins [file rspb20190470supp1.pdf]

Supplemental Figure 2

A

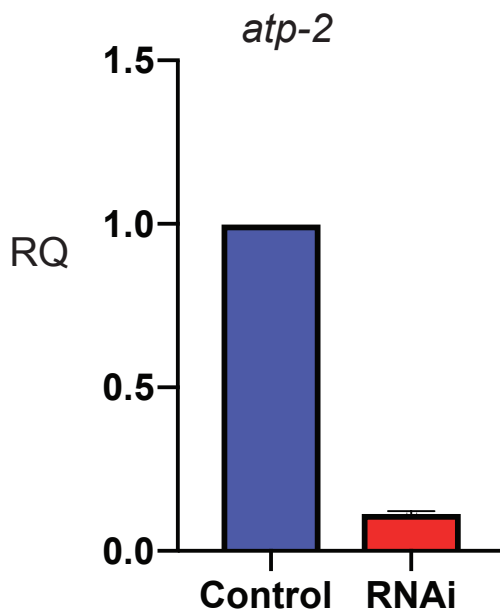

B

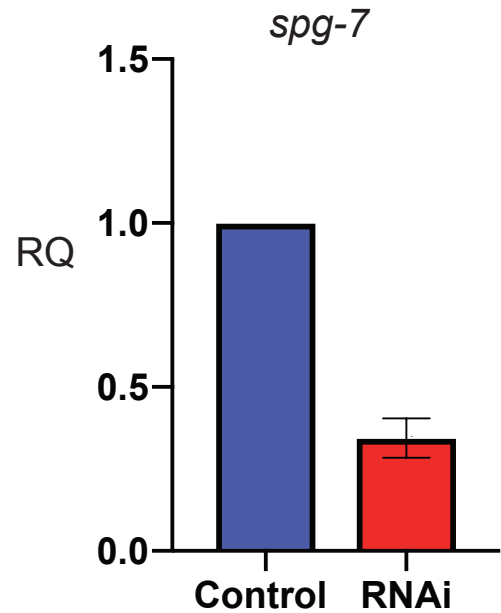

C

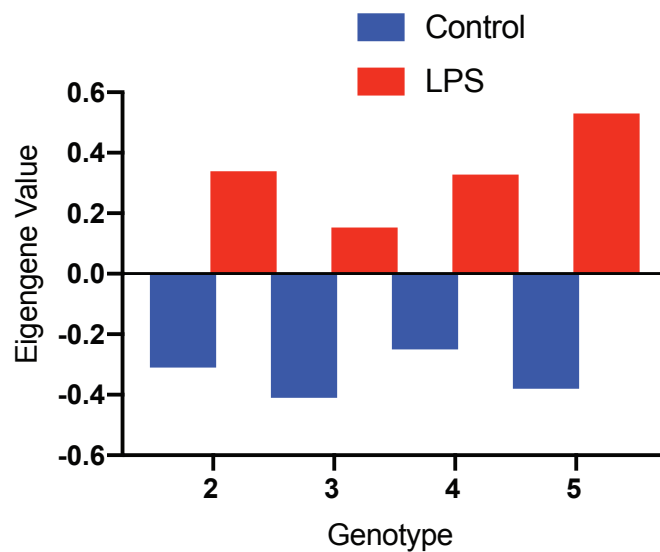

D

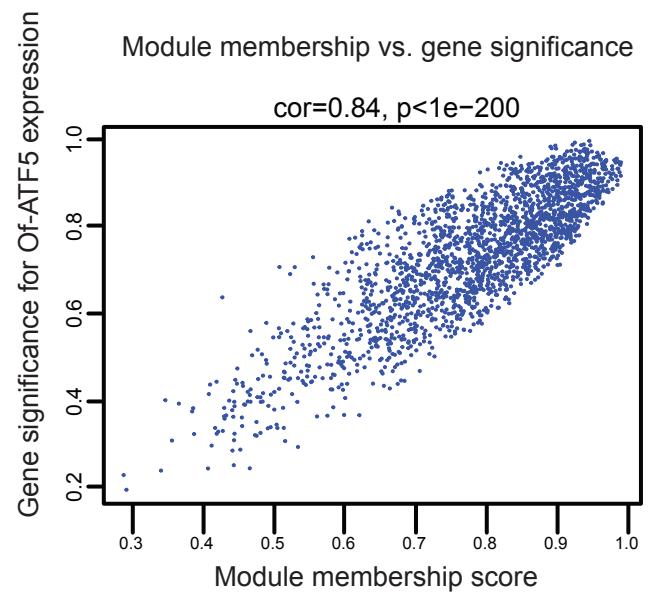

E

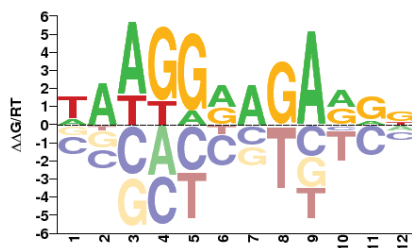

F

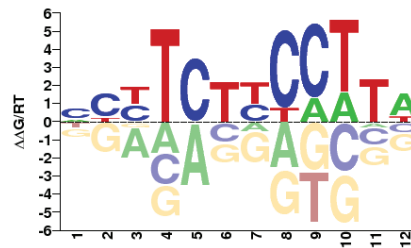

Supplement: Identifying components of the coral UPRmt [file rspb20190470supp2.pdf]
