# Supplementary material for: Uncovering a mitochondrial unfolded protein response in corals and its role in adapting to a changing world
Source: Proc Biol Sci. 2019 Jun 26;286(1905):20190470. doi: 10.1098/rspb.2019.0470 (PMC6599992; doi:10.1098/rspb.2019.0470)
Supplement: Supplementary legends [file rspb20190470supp6.docx]

**Supplemental Figure 1**: Homology of UPR^mt^ mediating proteins. (A) tblastn scores between the amino acid sequence of ATFS-1 and Hs-ATF5 against the *O. faveolata* genome. (B) Full amino acid sequence alignment between Of-ATF5, Hs-ATF5, and C. elegans ATFS-1. (*****) represents consensus between all three species, (•) represents amino acid similarity between two species. (C) Schematic of the transcriptional UPR^mt^ reporter *hsp-60_pr_::GFP* transgene construct being bound by ATFS-1/Of-ATF5. (D) Increased temperature induce the expression of two different UPR^mt^ reporter genes in *C. elegans* (*hsp-60_pr_::GFP, hsp-6_pr_::GFP*). (E) Quantification of the intensity of the fluorescent signal from panel D. (* p<0.01, one-way anova using the intensity in worms raised at 16 degrees as a reference).

**Supplemental Figure 2**: Identifying components of the coral UPR^mt^: (A-B) Level of gene depletion for worms raised on RNAi clones against atp-2 (87%, p < 0.0005)(A), or spg-7 (B) (77%, p<0.0005). Expression levels were calculated with the $\Delta\Delta$ Ct method, and normalized to Actin-3 for both genes. (C) Eigengene values of the highly correlated WGCNA module between genotype matched samples during treatment with LPS. (D) Scatterplot illustrating the module membership score plotted against the gene significance to Of-ATF5 for each contig in our highly correlated WGCNA module, with each dot representing a contig. (E-F) Schematic of the positional weighted matrix of Hs-ATF5 in the (C) forward direction and (D) reverse direction.

**Supplemental Table 1**: *O. faveolata* UPR^mt^ genes identified through WGCNA. Listed are the contigs with annotations found within the module significantly correlated to Of-ATF5 expression. Also listed are the gene significance correlation and P-values in relation to both Of-ATF5 expression, and module eigengene, as well as: uniport accession number, gene name and E-value. Contigs are sorted into functional categories based on manual annotations.

**Supplemental Table 2**: Genes from our WGNCA module with Hs-ATF5 binding sites within their promoters. Listed are contig ID, binding location in the scaffold, strand oritentation, pvalue, matched sequence, NCBI ID and protein name.

**Supplemental Table 3:** Primers used for qPCR reactions.
